# RETRACTION: Green Fabrication of Silver Nanoparticles Using *Euphorbia Serpens* Kunth Aqueous Extract, their Characterization, and Investigation of its *in Vitro* Antioxidative, Antimicrobial, Insecticidal, and Cytotoxic Activities

**DOI:** 10.1155/bmri/9790540

**Published:** 2026-06-30

**Authors:** BioMed Research International

RETRACTION: N. Ahmad, Fozia, M. Jabeen, Z. U. Haq, I. Ahmad, A. Wahab, Z. U. Islam, R. Ullah, A. Bari, M. M. Abdel‐Daim, F. M. El‐Demerdash, M. Y. Khan, “Green Fabrication of Silver Nanoparticles using *Euphorbia serpens* Kunth Aqueous Extract, Their Characterization, and Investigation of Its *In Vitro* Antioxidative, Antimicrobial, Insecticidal, and Cytotoxic Activities,” *BioMed Research International*, 2022, 5562849, https://doi.org/10.1155/2022/5562849

The above article, published online on 10 January 2022 in Wiley Online Library (wileyonlinelibrary.com), has been retracted by agreement between the journal Editor‐in‐Chief, Yoel Klug; and John Wiley & Sons Ltd. The retraction has been agreed due to concerns identified with Figure 3 and other data in the article, as reported on PubPeer [1]. Specifically:

• After adjusting the aspect ratio, Figure 3 appears highly similar to the image shown in Figure 5 of [2], an article from different authors
• In Figures 6, 7, 8 and 9, all of the error bars appear to be identical

The authors were contacted about the concerns raised. After assessment of the response of the authors by the editor, the results and conclusions presented are no longer deemed reliable, and the editor has lost confidence in the results and conclusions published. It is therefore retracted.

Dr Ijaz Ahmad has stated in their response to the concerns that the following authors solely contributed to reviewing the final draft of the manuscript.

• Musarrat Jabeen, Zia Ul Haq and Fozia

Additionally, the corresponding authors also stated that the following authors solely contributed to the funding of the study:

• Muhammad Yahya Khan, Fatma M. El‐Demerdash, Mohamed M. Abdel‐Daim, Ahmed Bari and Riaz Ullah

Ijaz Ahmad disagrees with the retraction, whilst the other authors remained unresponsive when informed of the retraction.
